# Case Report: Complete atrioventricular block and left ventricular outflow tract obstruction as primary manifestations of immunoglobulin G4-related disease

**DOI:** 10.3389/fcvm.2025.1544902

**Published:** 2025-06-19

**Authors:** Yihua Liu, Lucie Schnedecker, Florian Eggenspieler, Damien Mandry, Shirine Mohamed, Daniel Grandmougin, Juan Pablo Maureira

**Affiliations:** ^1^ Department of Cardiovascular Surgery and Heart Transplantation, University Hospital of Nancy, University of Lorraine, Vandoeuvre-lès-Nancy, France; ^2^ Department of Anatomo-Pathology, University Hospital of Nancy, University of Lorraine, Vandoeuvre-lès-Nancy, France; ^3^ Department of Cardiology, University Hospital of Nancy, University of Lorraine, Vandoeuvre-lès-Nancy, France; ^4^ Department of Radiology, University Hospital of Nancy, University of Lorraine, Vandoeuvre-lès-Nancy, France; ^5^ Department of Immunology, University Hospital of Nancy, University of Lorraine, Vandoeuvre-lès-Nancy, France

**Keywords:** immunoglobulin G4, cardiac skeleton, atrioventricular block, valvular prosthesis, glucocorticoids

## Abstract

**Background:**

Immunoglobulin G4-related disease (IgG4-RD) is a systemic immunologic fibroinflammatory condition that can affect a wide range of organ systems. In this study, we present a rare case in which the primary manifestations were complete heart block and left ventricular outflow tract (LVOT) obstruction due to the involvement of the cardiac skeleton.

**Case presentation:**

A 66-year-old man presented with syncope. An evaluation revealed paroxysmal complete atrioventricular block (AVB) and LVOT obstruction caused by diffuse fibrous thickening of the aortic valve extending to the anterior mitral leaflet, with a maximal gradient of 70 mmHg. A semiurgent operation was performed, including aortic and mitral valve replacement with bioprostheses and implantation of a definitive epicardial pacemaker. A histopathologic examination suggested IgG4-RD; however, glucocorticoid therapy was initially withheld. Two months later, the patient developed recurrent AVB and pacemaker dysfunction. Salvage glucocorticoid therapy led to the normalization of pacemaker thresholds.

**Conclusions:**

IgG4-RD may underlie valvulopathy and conduction disorders via lymphoplasmacytic infiltration and fibrosis of the cardiac skeleton. Surgical intervention and timely glucocorticoid therapy are associated with favorable outcomes.

## Background

Immunoglobulin G4-related disease (IgG4-RD) was first recognized as a systemic immunologic fibroinflammatory condition in the year 2003. It is characterized histologically by lymphoplasmacytic infiltration and tumorous lesions. Although IgG4-RD has been described in nearly every organ system, cardiac involvement manifesting as atrioventricular block (AVB) and left ventricular outflow tract (LVOT) obstruction is extremely rare. Table 1 presents the timeline.

**Table 1:** Timeline.

Day	Events
1	Patient admitted with syncope; echocardiography showed thickened aortic cusps extending to the mitral valve with LVOT obstruction; Holter ECG revealed a paroxysmal complete AVB. Semiemergent surgery was indicated.
3	Aortic and mitral valves replaced with bioprostheses; epicardial electrodes and pacemaker implanted.
10	Discharged with an uneventful postoperative course.
24	Pathology suggested IgG4-RD.
30	Admitted for IgG4-RD workup; serum IgG4 elevated but below diagnostic threshold; PET scheduled; glucocorticoids withheld.
62	Another syncope; pacemaker dysfunction with high stimulation thresholds discovered.
65	Readmitted; PET scan advanced; glucocorticoid therapy initiated.
77	Electrophysiological control showed improved pacing thresholds.
278	Epicardial electrode function normalized.
312	Patient doing well, prednisolone discontinued.

## Case presentation

A 66-year-old man presented with syncope. He had no significant past medical history except for active tobacco use and no relevant family history. On examination, he had a blood pressure of 109/55 mmHg, heart rate of 74 beats per minute, periorbital ecchymosis due to trauma from syncope, and a systolic murmur in the second intercostal space.

**Figure 1:**
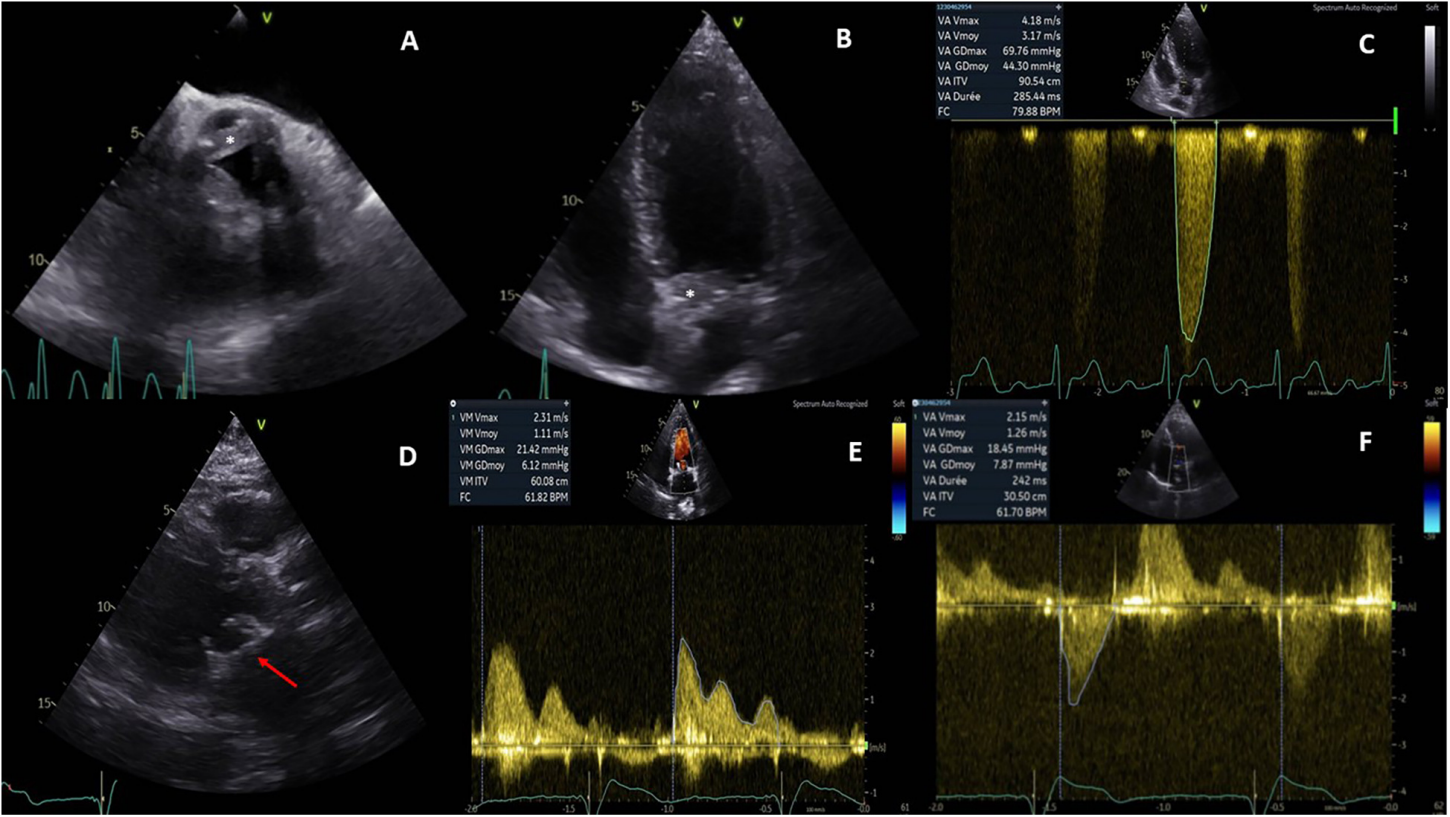
Pre- and postoperative echocardiography. Preoperative echocardiography showed an abnormal thickening of aortic cusps (*, **A)** extending to the anterior leaflet of the mitral valve (*, **B)** and resulting a significant subaortic obstruction **(**maximal gradient of 70 mmHg, **C)**. Postoperative echocardiography demonstrated a well-positioned mitral bioprosthesis **(**arrow, **D)**; the mean transprosthetic gradient was 1.1 mmHg **(E)** in the mitral position and 7.8 mmHg **(F)** in the aortic position.

**Figure 2:**
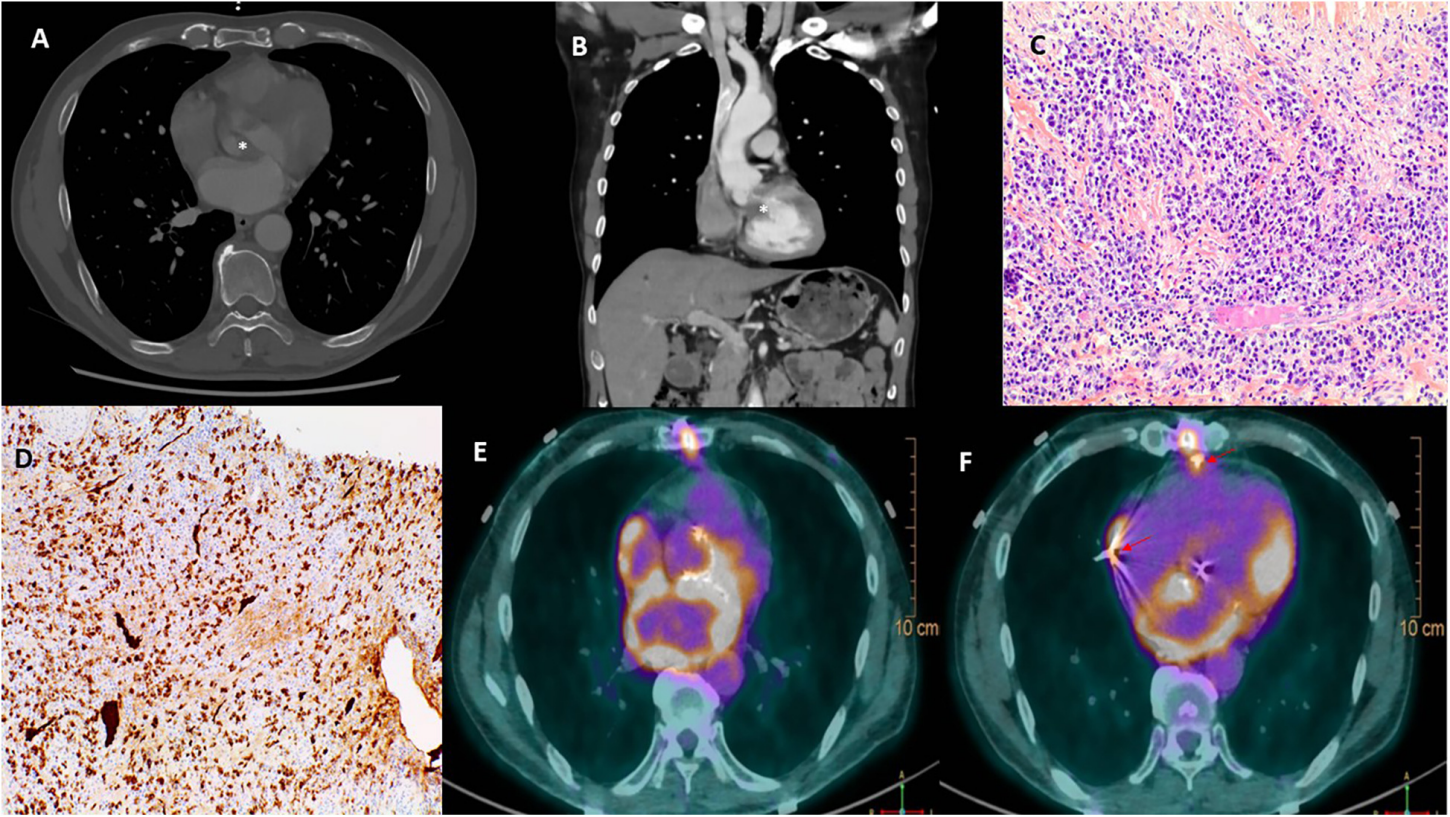
Multimodal images depicting IgG4-RD. Computerized tomography confirmed a significant thickening of the aortic valve (*, **A)** and an obstruction of the left ventricular outflow tract (*, **B)**. Hematoxylin and eosin stain **(C)** of resected tissue demonstrated storiform fibrosis and dense inflammatory infiltrate with a majority of lymphocytes and plasma cells. Immunoglobulin G4 immunohistochemical stain **(D)** showed numerous IgG4+ plasma cells. ^18^F-Fluorodeoxyglucose positron emission tomography **(E,F)** demonstrated a hypermetabolism due to a recent surgery in the left atrium, the aortic root, and the sites of epicardial electrodes **(**arrow, **F)**.

Initial electrocardiogram (ECG) showed sinus rhythm with right bundle branch block and left anterior fascicular block. Holter monitoring revealed paroxysmal complete AVB lasting 8 s. Transthoracic and transesophageal echocardiography revealed preserved left ventricular ejection fraction (LVEF, 68%), moderate aortic regurgitation, and significant thickening of the aortic valve extending to the aorto-mitral curtain and anterior mitral valve (Figures 1A,B), causing LVOT obstruction with maximal and mean gradient of 70 and 44 mmHg (Figure 1C), respectively. Computerized tomography (CT) confirmed hyperplasic tissue at the LVOT (Figures 2A,B). Coronary angiography showed normal results. Laboratory tests showed mild leucocytosis (135 000/ml), slightly elevated C-reactive protein (12 mg/L), and NT-pro brain natriuretic peptide (295 pg/ml); troponin level was normal.

The differential diagnosis included subacute bacterial endocarditis and inflammatory pseudotumor involving the aortic and mitral valves. A semiurgent operation was performed to replace both valves and implant an epicardial pacemaker. Intraoperatively, the aortic leaflets were fibrotic and thickened (8 mm) without calcification or vegetation. Aortic and mitral bioprostheses were implanted, and the LVOT was widened by shaving the aorto-mitral curtain.

The postoperative course was uneventful, and the patient was discharged on day 10. Echocardiography showed well-functioned aortic and mitral prostheses (Figures 1D–F); electrophysiological control of epicardial electrodes and pacemaker was normal. A pathological examination revealed lymphoplasmacytic infiltration and IgG4+/IgG+ cell ratio of 30%–50% (Figures 2C,D), suggesting IgG4-RD.

The patient was then referred to immunologists for the workup of IgG4-RD. The serum IgG4 concentrations were elevated (highest 133 mg/dl) but below the diagnostic cutoff (135 mg/dl). The diagnosis of malignant hemopathies, vasculitis, sarcoidosis, and other autoimmune diseases such as Sjögren's syndrome were also excluded. Since no other organ involvement was found, steroids were withheld and a positron emission tomography (PET) scan was scheduled.

Two months later, the patient had another syncopal episode. ECG showed complete AVB and pacemaker dysfunction; PET scan revealed hypermetabolism in the aortic root, left atrium, and epicardial electrode sites, while the interpretation was difficult due to recent surgery (Figures 2E,F). Glucocorticoids (intravenous loading dose of methylprednisolone 750 mg, followed by prednisolone 60 mg/day) were started.

Two weeks later, pacing thresholds improved significantly; 9 months later, they were normalized, avoiding pacemaker revision. At 1 year, the patient remained stable, and prednisolone was discontinued after tapering.

## Discussion

IgG4-RD is a systemic fibroinflammatory disease marked by IgG4+ plasma cell infiltration and storiform fibrosis (1). The diagnosis relies mainly on histopathological analysis with quantitative criteria such as ratio of IgG4-positive plasma cells/IgG-positive cells greater than 40% and the number of IgG4-positive plasma cells greater than 10 per high-powered field. Even though approximately 30% of patients have normal serum IgG4 concentrations, the 2020 revised comprehensive diagnostic criteria for IgG4-RD adopted a serum IgG4 cutoff value of 135 mg/dl (2). In our case, the serum IgG4 concentration was elevated but never reached the cutoff level, and the ratio of IgG4+/IgG+ plasma cells was not consistently greater than 40% in different microscopic fields. Steroids were withheld for the characteristics a probable instead of definite diagnosis of IgG4-RD; another reason was to avoid secondary effects related to glucocorticoid therapy such as hypertension, electrolyte disorders, wound infection, and so on.

Cardiovascular involvement in IgG4-RD is rare but increasingly reported, affecting coronary arteries, heart valves, myocardium, pericardium, aorta, and peripheral vessels (3). The diagnosis is difficult due to non-specific imaging and limitations in obtaining tissue samples. It might be warranted to measure the serum concentration of IgG4 in patients presenting with unexplained valvular tumorous lesions. In case of valve destruction due to lymphoplasmacytic infiltrate, valve replacement with prostheses might be the only solution; periprosthetic leakage could be an issue in IgG4-RD, while preoperative and/or adjuvant glucocorticoid therapy might reduce this risk. Kosugi et al*.* (4) reported a rare case of IgG4-RD with evolving aortic regurgitation under intensive steroid therapy. They believed that preoperative glucocorticoid therapy was mandatory to avoid perivalvular leakage, despite its potential risk of exacerbating aortic regurgitation and heart failure. However, in the case of our patient, effective steroid therapy was started 2 months after valvular surgery and lasted 8 months. The echocardiography of control showed no periprosthetic leakage. Indeed, further multimodal imaging follow-up is warranted to detect this severe complication.

Evolving AVB and secondary pacemaker dysfunction are notable in our patient. There are two possible explanations: a tiny shift of the epicardial electrode or IgG4-RD-induced local inflammatory activity. Even though the patient responded well to delayed steroid therapy, the precise pathophysiology of pacemaker dysfunction in IgG4-RD remains unclear, since the fibroinflammatory process seemed to be confined to the cardiac skeleton without signs of extension to the myocardium or pericardium. Could the mechanical injury related to epicardial electrode provoke a lymphoplasmacytic infiltration through some non-autoimmunologic mechanisms?

Glucocorticoids are generally accepted as the first-line treatment; however, there is no consensus on the optimal duration, which varies from 3 months to 3 years (1, 5). We need to balance the benefit-risk ratio between disease relapse and secondary effects of long-term glucocorticoid therapy. The difficulty lies in the lack of reliable biomarkers predicting the recurrence of IgG4-RD. In most patients, serum IgG4 concentration decreases but remains above normal values with glucocorticoid treatment; in some patients, the disease remains in remission despite persistent elevations of serum IgG4 levels, whereas disease relapse occurs in about 10% of patients with normal IgG4 concentrations (6). Thus, a close clinical follow-up and multimodal imaging is critical to detect disease relapse.

## Conclusion

In conclusion, this case highlights a rare presentation of IgG4-RD with LVOT obstruction and AV block, treated with valve replacement and pacemaker implantation. Postoperative pacemaker dysfunction was successfully managed with delayed glucocorticoid therapy.

## Data Availability

The original contributions presented in the study are included in the article/Supplementary Material, further inquiries can be directed to the corresponding author.
